# Seroprevalence of measles antibody among immigrants in Gwangju, South Korea

**DOI:** 10.3389/fpubh.2024.1505489

**Published:** 2024-12-19

**Authors:** Ran Lee, Sarah Kim, Hyerin Na, Ji In Seo, Jang Gwon Yoon, A Ram Park, So Hyun Bae, So Yeong Park, Jun Hwi Cho, Jin Kim, Seong-Woo Choi, Sun-Seog Kweon, Bongkyu Sun, Kyunghak Kim, Kyung-Hwa Park, Seong Eun Kim

**Affiliations:** ^1^Gwangju Center for Infectious Diseases Control and Prevention, Gwangju, Republic of Korea; ^2^Department of Public Health, Graduate School, Chonnam National University, Gwangju, Republic of Korea; ^3^Department of Infectious Diseases, Chonnam National University Medical School, Gwangju, Republic of Korea; ^4^Department of Public Health, Graduate School of Health Science, Chosun University, Gwangju, Republic of Korea; ^5^Department of Health Science, Graduate School of Chosun University, Gwangju, Republic of Korea; ^6^Department of Preventive Medicine, Chosun University Medical School, Gwangju, Republic of Korea; ^7^Department of Preventive Medicine, Chonnam National University, Hwasun, Republic of Korea; ^8^Center for Global Diaspora Studies, Chonnam National University, Gwangju, Republic of Korea

**Keywords:** measles, seroprevalence, antibody, immigrant, South Korea

## Abstract

**Introduction:**

Measles remains a public health concern, particularly among populations with suboptimal vaccination coverage, including immigrants. Understanding the seroprevalence of measles antibodies in immigrant populations is essential to inform tailored vaccination strategies and reduce the risk of measles reintroduction.

**Methods:**

This study evaluated measles IgG seroprevalence among 651 immigrants from 30 countries residing in Gwangju, South Korea. Participants were recruited between September 2022 and August 2024, and measles antibody levels were assessed using the LIAISON® XL assay. Statistical analyses included Fisher’s exact and chi-squared tests to identify associations between seropositivity and demographic factors.

**Results:**

Measles antibody positivity rates varied significantly by age group (*p* < 0.01). Individuals born after 1995 exhibited the lowest seroprevalence (63.7%), while those born in 1964 or earlier were all seropositive (100%). Seroprevalence was particularly low among immigrants from Russia (68.1%), Kazakhstan (70.6%), Ukraine (72.7%), Mongolia (75.5%), and Cambodia (78.1%). Long-term residents demonstrated higher antibody positivity (92.9%) than other visa categories (*p* < 0.01), and women had higher seropositivity (85.8%) compared to men (80.0%), with a near-significant difference (*p* = 0.05).

**Discussion:**

The higher seropositivity observed among long-term residents and women is likely due to prior immunization with the MMR (Measles Mumps Rubella) vaccine, which is recommended to prevent congenital rubella syndrome as part of pre-pregnancy vaccination protocols. This study underscores the importance of implementing tailored vaccination programs based on the characteristics of immigrant populations and focusing on countries with low seroprevalence to effectively prevent measles reintroduction.

## 1 Introduction

Measles is a highly contagious viral disease, preventable through effective vaccination. Despite global efforts to eradicate measles, the disease remains a significant public health threat, particularly in regions with suboptimal vaccination coverage. The World Health Organization (WHO) declared the elimination of measles in South Korea in 2014, a testament to the country’s successful vaccination programs. However, South Korea has continued to experience sporadic outbreaks due to imported cases (1, 2), as measles can be easily transmitted by individuals traveling internationally without adequate vaccination or whose immunity has waned over time.

Given the importance of maintaining high levels of immunity within the population to prevent outbreaks, it is crucial to assess the susceptibility of immigrants to measles and other vaccine-preventable diseases. Immigrant populations may have varying levels of immunity depending on their country of origin, access to healthcare, and history of vaccination. Understanding the seroprevalence of measles antibodies among these groups is essential for informing vaccination policies and ensuring adequate protection against potential outbreaks.

This study aims to evaluate the seroprevalence of measles antibodies among immigrants in Gwangju Metropolitan City, South Korea and provide insights to inform vaccination strategies. This research could contribute to strengthening public health policies by identifying gaps in immunity and proposing effective interventions for preventing the spread of measles among immigrants and the broader population.

## 2 Methods

This study was conducted among migrants living in Gwangju, South Korea, from September 2022 to August 2024. Each phase included a one-month pre-promotion period, followed by 2 to 3 months of implementation annually: September to October in 2022, October to November in 2023, and June to August in 2024. The study was publicized through posters and placards in English, Russian and Korean, as well as through the ethnic local community. Participants were asked to give informed consent, after which blood samples were taken, and sociodemographic information was collected through questionnaires in the above languages (Supplementary materials). Sociodemographic information including country of origin, birth year, sex, smoking, self-perceived health status, unmet medical needs within the past year, national health insurance, occupation type, income, visa status, and vaccination history (self-reporting) were collected via a questionnaire. These variables were categorized as follows: Birth year (1964 and before, 1965–1984, 1985–1994, or 1995 and after), gender (male or female), country of origin (e.g., Vietnam, Uzbekistan, China, Cambodia, Mongolia, or other), smoking status (current smoker, quit or never smoking), self-perceived health status (very good, good, fair, poor or very poor), unmet medical needs within the past year (yes, no, or not answered), national health insurance coverage (yes or no), occupation type (permanent worker [full-time], irregular worker [part-time/temporary, self-employed, or not working]), monthly income (less than 1.5 million KRW, 1.5–2.5 million KRW, 2.5–3.5 million KRW, more than 3.5 million KRW, or no personal income), visa status (long-term residential immigrants: permanent resident [F5], marriage migrant [F6], or Korean citizenship; other visa types: overseas ethnic Koreans [F4], working visit [H2], family visit [F1], resident [F2], non-professional employment [E9], study [D2], undocumented, or refused to reveal), vaccination history (yes, no, or do not know).

Participants underwent blood and urine tests, including measles immunoglobulin G (hereafter, measles IgG), as well as antibody tests for infectious diseases, and complete blood count, aspartate transaminase and alanine transaminase, creatinine, bilirubin, hemoglobin A1c, and a chest X-ray. All clinical laboratory tests except chest X-ray and urinalysis were performed by Seegene (Seegene, Seoul, Korea). Serum levels of measles IgG were evaluated using LIAISON® XL (DiaSorin, Dietzenbach, Germany). Measles IgG levels greater than 16.5 AU/mL were positive and considered protective, with levels between 13.5–16.4 AU/mL as equivocal, and less than 13.5 AU/mL as negative, according to the manufacturer’s guidelines. The LIAISON® XL assay demonstrates a specificity of 97.4% and a sensitivity of 94.7% for detecting measles IgG (manufacturer’s data). Seroprevalence was defined as the proportion of participants with measles IgG levels above the protective threshold of 16.5 AU/mL, expressed as a percentage of the total number of participants tested. In this study, the terms measles seroprevalence and measles IgG positive rate are used interchangeably to represent the same concept. Participants received medical consultation, and measles immunization was provided based on serological results, if necessary.

All statistical analyses were conducted using SPSS version 27.0 (IBM Corp, Armonk, NY). Continuous variables were assessed using the Mann–Whitney U-test or Student’s t-test depending on data distribution. Categorical variables were assessed using the Pearson χ2 test or Fisher’s exact test. Variables were considered statistically significant if the *p*-value was less than 0.05. This study protocol was approved by the Institutional Review Board (IRB) of Chonnam National University Hospital (CNUH 2022–365). The IRB confirmed informed consent.

## 3 Results

A total of 651 participants took part in the study, representing the following countries of origin: Vietnam 104 (16%), Uzbekistan 75 (11.5%), China 65 (10%), Mongolia 53 (8.1%), Kazakhstan 51 (7.8%), Russia 47 (7.2%), Philippines 43 (6.6%), Nepal 35 (5.4%), Ukraine 22 (3.4%), India 13 (2.0%), Bangladesh 9 (1.4%), Kyrgyzstan 8 (1. 2%), Nigeria, Liberia, and Indonesia each had 7 (1.1%) participants. Cameroon, Kenya, and Thailand each had 5 (0.8%) participants, Ghana and Sri Lanka each had 4 (0.6%) participants, Japan, Myanmar, and Pakistan each had 3 (0.5%) participants, Mexico 2 (0.3%), Uganda, France, South Africa, Italy, Laos, Canada, and Malaysia each had 1 participant (0.2%). The median age of participants was 36 years (IQR 28–48), with 285 (43.8%) men and 366 (56.2%) women.

Measles IgG seropositivity rates by year of birth group and nationality of participants are shown in Table 1. All participants born in 1964 or earlier were measles IgG positive, and there was a clear decreasing trend in measles IgG positivity rate in more recent birth year group across all countries. Table 1 also includes the measles IgG antibody positivity rates by vaccination history for each country of origin. Vaccinated participants generally exhibited higher seropositivity rates compared to unvaccinated participants, with notable variability across countries. Vietnamese vaccinated individuals having a seropositivity rate of 88.2%, while Russia showed a lower rate at 66.7%. Among unvaccinated participants, Cambodia’s group showed a seropositivity rate of 77.8%, indicating variability in immunity levels by vaccination status and country of origin.

**Table 1:** Number of immigrants who participate in this study according to country of origin, birth cohort and gender.

	Country of origin										
	Vietnam	Uzbekistan	China	Cambodia	Mongolia	Kazakhstan	Russia	Philippines	Nepal	Ukraine	Others
Total	104	75	65	64	53	51	47	43	35	22	92
Birth cohort											
1964 and before	18(100)	22(100)	9(100)	1(100)	3(100)	8(100)	12(100)	1(100)	0(−)	6(100)	5(100)
1965–1984	41(100)	25(92)	34(88.2)	8(75)	7(85.7)	14(92.9)	13(61.5)	25(100)	5(100)	4(75)	26(96.2)
1985–1994	27(100)	11(72.7)	18(88.9)	35(82.9)	18(94.4)	8(50)	10(60)	8(100)	19(89.5)	6(50)	33(84.8)
1995 and after	18(66.7)	17(70.6)	4(100)	20(70)	25(56)	21(52.4)	12(50)	9(66.7)	11(54.5)	6(66.7)	28(71.4)
Gender											
Male	37(89.2)	23(73.9)	17(88.2)	41(75.6)	17(76.5)	19(52.6)	12(50)	21(90.5)	27(77.8)	8(87.5)	63(88.9)
Female	67(97)	52(92.3)	48(91.7)	23(82.6)	36(75)	32(81.3)	35(74.3)	22(95.5)	8(87.5)	14(64.3)	29(75.9)
Vaccination history											
Yes	17(88.2)	64(85.9)	28(89.3)	1(100)	18(72.2)	37(81.1)	39(66.7)	6(83.3)	5(100.0)	21(71.4)	22(86.4)
No	45(100)	7(85.7)	12(91.7)	45(77.8)	16(87.5)	8(62.5)	3(100)	27(92.6)	12(83.3)	0(0)	47(91.5)
Do not know	42(90.5)	4(100.0)	25(92.0)	18(77.8)	19(68.4)	6(16.7)	5(60.0)	10(100)	18(72.2)	1(100)	23(69.6)

Parentheses indicate measles seropositive rate of each group.

**Figure 1:**
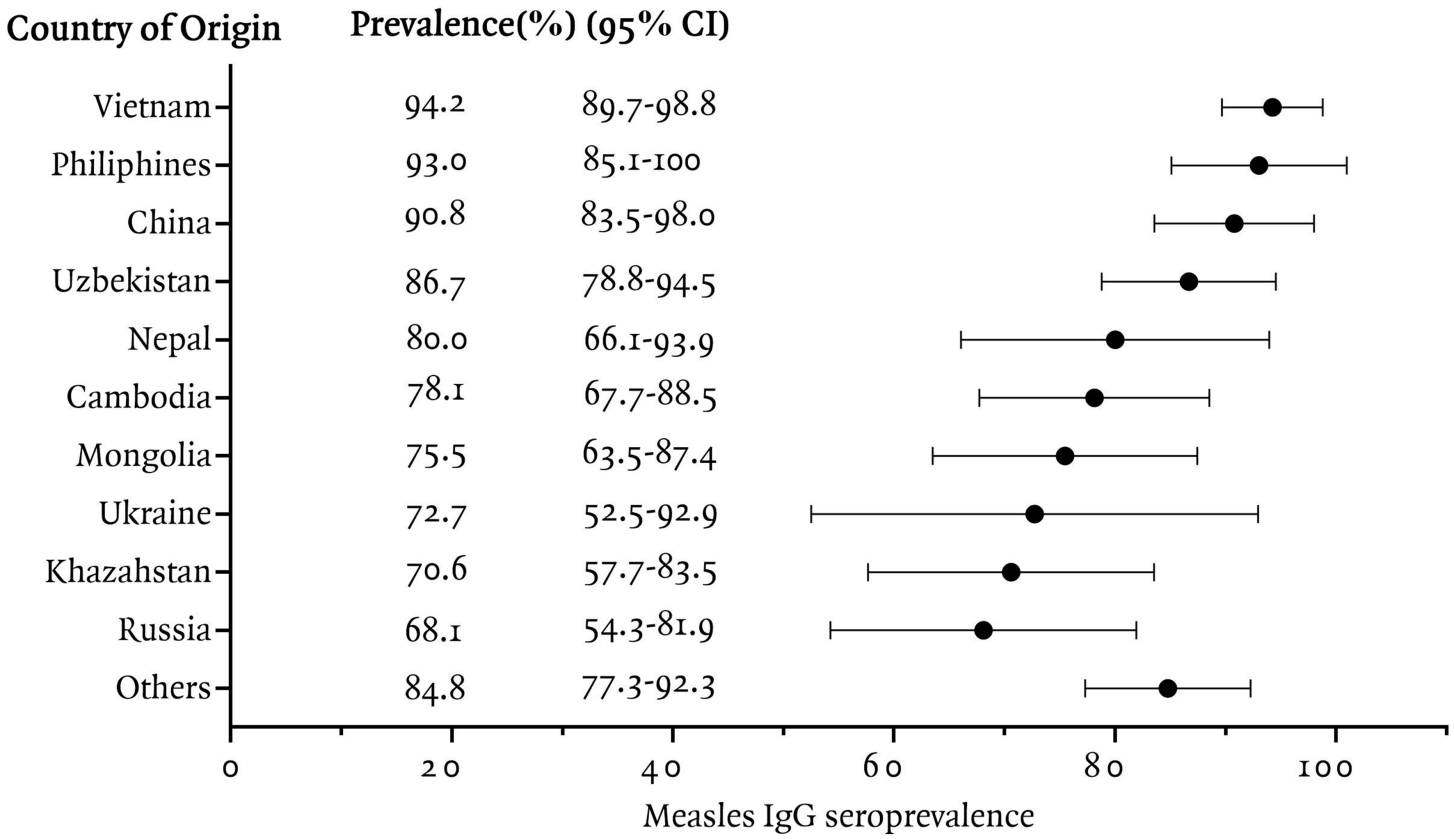
Measles IgG seroprevalence among immigrants residing in Korea, categorized by country of origin.

**Figure 2:**
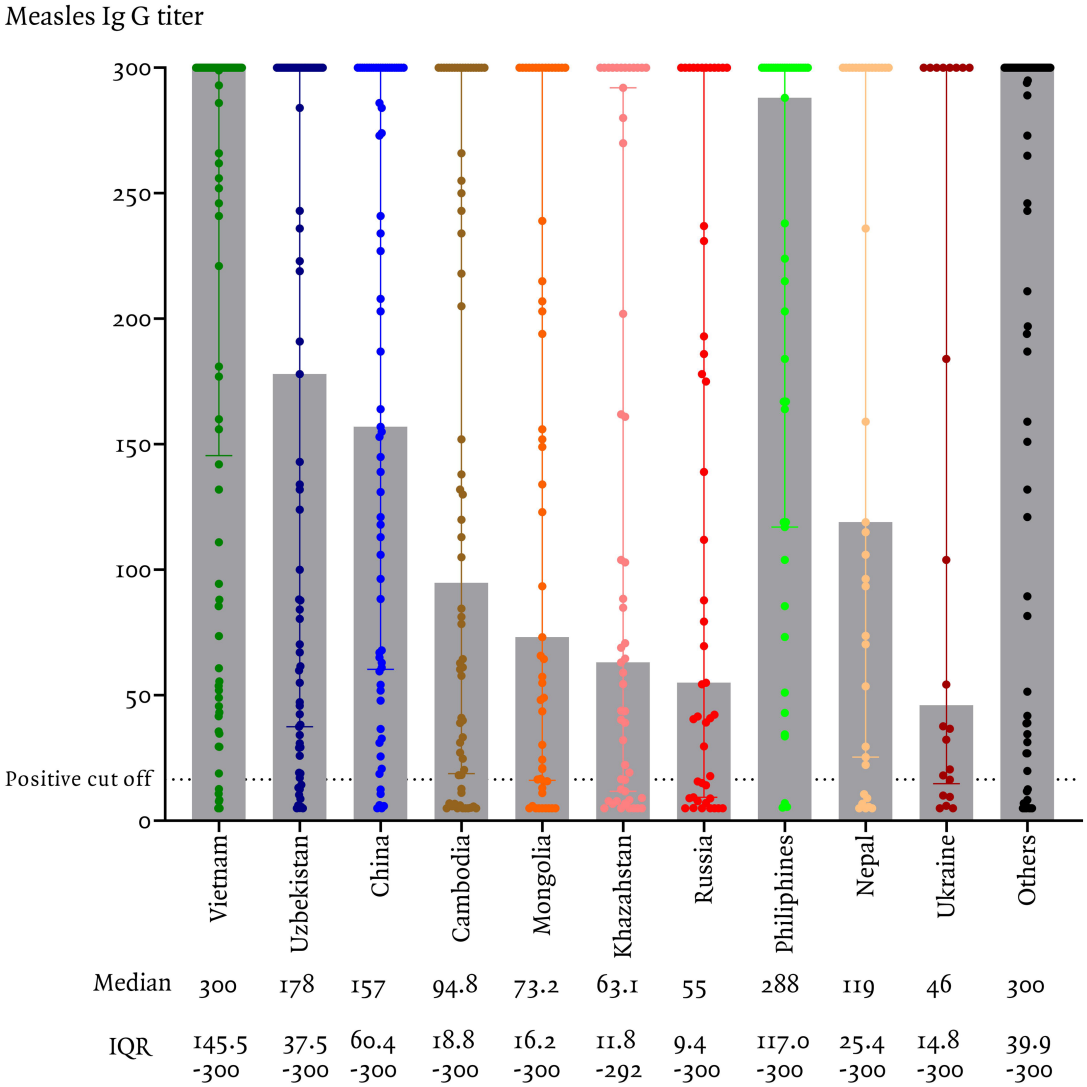
Measles IgG titers among immigrants residing in Korea, categorized by country of origin.

In this study, measles seroprevalence by country of origin was greater than 90% in Vietnam, the Philippines, and China, between 80 and 90% in Uzbekistan, Nepal, and other countries, and less than 80% in Cambodia, Mongolia, Ukraine, Kazakhstan, and Russia (Figure 1). The measles Ig G titer distribution for each country is presented, Figure 2 illustrating the median and interquartile range (IQR) values for participants by their country of origin. The titer levels indicate variability across countries, with lower titers observed in participants from Russia, Kazakhstan, Mongolia, Cambodia, and Ukraine, which aligns with their lower seroprevalence rates.

We compared antibody positivity according to socio-demographic characteristics of immigrants (Table 2). We categorized participants by year of birth into four groups and found that recent births were associated with lower measles IgG positivity rates (*p* < 0.01, chi-squared test). In addition, there was a statistical difference in measles IgG seroprevalence rate between long-term residents (marriage visa, permanent residence, Korean citizenship) and other immigrants (*p* = 0.01, Chi-square test). Other factors such as national insurance coverage, monthly income, occupation type and unmet medical needs did not statistically differ measles IgG seropositivity in immigrants.

**Table 2:** Demographic and socioeconomic characteristics with measles seroprevalence in immigrants residing South Korea.

	Seropositive	Seronegative or equivalent	*p* value
Birth year			<0.01*
Born in 1964 or earlier	85 (100)	0 (0)	
1965–1984	185 (91.6)	17 (8.4)	
1985–1994	163 (84.5)	30 (15.5)	
Born in 1995 or later	109 (63.7)	62 (36.3)	
Sex			0.05
Male	228 (80.0)	57 (20.0)	
Female	314 (85.8)	52 (14.2)	
Smoking			0.28
Current smoker	85 (81.0)	20 (19.0)	
Quit or never smoking	446 (85.1)	78 (14.9)	
Self-perceived health status			0.70
Very good	33 (78.6)	9 (21.4)	
Good	132 (86.3)	21 (13.7)	
Fair	309 (84.0)	59 (16.0)	
Poor	55 (85.9)	9 (14.1)	
Very poor	3 (100)	0	
Unmet medical needs within 1 year^a^			0.08
Yes (at least once)	268 (87.0)	40 (13.0)	
No	262 (81.9)	58 (18.1)	
Not answered	12 (52.2)	11 (47.8)	
National health insurance coverage			0.63
Yes	345 (82.7)	72 (17.3)	
No	197 (84.2)	37 (15.8)	
Occupation type			0.79
Permanent worker (full-time)	231 (82.8)	48 (17.2)	
Irregular worker (part time/temporary)	36 (85.7)	6 (14.3)	
Self-employed	4 (100)	0 (0)	
Not working	271 (83.1)	55 (16.7)	
Monthly personal income			0.35
Below 1,500,000 won	134 (86.5)	21 (13.5)	
1,500,000–2,500,000 won	138 (85.2)	24 (14.8)	
2,500,000–3,500,000 won	65 (77.4)	19 (22.6)	
Above 3,500,000 won	15 (88.2)	2 (11.8)	
No personal income	190 (81.5)	43 (18.5)	
Visa status			<0.01*
Long-term residential immigrants^b^	91 (92.9)	7 (7.1)	
Other visa/refused to reveal^c^	451 (81.6)	102 (18.4)	

*Statistical significance (*p* < 0.05, Chi-square test).

a Unmet medical needs within 1 year were compared using two categories: ‘yes (at least once)’ and ‘no’.

b Long-term residential immigrants include 21 permanent resident visa (F5), 50 marriage visa (F6) and 27 cases of naturalization (Korean citizen). Ninety-three was female of the 98 participants with total long-term residential immigrants.

c There were 142 cases of undocumented or refused to reveal.

## 4 Discussion

In this study, we evaluated measles IgG seroprevalence among 651 immigrants from 30 countries residing in Gwangju, South Korea. The results revealed significant variability in seropositivity based on country of origin and age. Across all countries, younger age groups consistently exhibited lower measles antibody positivity rates, which is in line with similar findings from previous research (3, 4). Participants born in 1984 or later showed a measles IgG positivity rate of 74.7%, whereas all participants born in 1964 or earlier were positive. The low positivity rate in younger age groups is thought to be due to the vaccine-induced immunity waning over time, but the natural immunity following measles infection is maintained in older age groups (5).

The study identified particularly low measles IgG positivity rates in immigrants from Russia (68.1%), Kazakhstan (70.6%), Ukraine (72.7%), Mongolia (75.5%), and Cambodia (78.1%). Russia has maintained high measles vaccination coverage rates since introducing a two-dose schedule in 1986 (6). Measle Containing Vaccine 1 (MCV1) coverage increased steadily from 76% in 1986 to over 90% by 1994, and since 1996, Russia has consistently maintained an MCV1 coverage rate exceeding 95% (7). However, gaps in immunity may exist among immigrants due to the waning of vaccine-induced immunity over time, as immunity from vaccination tends to decline more quickly compared to that acquired through natural infection (8). A prior study in Russia reported that 21.4–22.7% of healthcare workers and women in labor aged 21–43 years were seronegative (9). Given that the previous study population was likely to have received additional Measles, Mumps, Rubella (MMR) vaccination to prevent hospital infections and congenital rubella syndrome, the measles IgG positivity rate among Russian immigrants in our study may better reflect the actual immunity status of the general population.

Kazakhstan, like Russia, has maintained over 90% MCV1 coverage since the 1990s (10). Notably, reported vaccination rates for MMR in Kazakhstan were exceptionally high, with MCV1 coverage reaching 98% in 2013 and exceeding 99% in 2014 and 2016. These rates theoretically suggest sufficient population immunity to prevent measles outbreaks. However, our study revealed a measles IgG positivity rate of only 70.6% among Kazakhstan immigrants. A possible explanation lies in findings from a 2015 measles outbreak in Kazakhstan, which revealed that 27% of confirmed cases reported no prior vaccination, and nearly half (46.7%) lacked documented vaccination records (10). This discrepancy suggests that reported vaccination rates may not fully reflect actual vaccination practices, highlighting potential gaps in vaccine delivery or documentation. These findings underscore that even in countries with reported high vaccination rates, large number of individuals who unverified vaccination histories and inconsistent record-keeping may be susceptible to measles.

Ukraine exhibited fluctuating vaccination coverage, which likely contributed to its lower seroprevalence (72.7%). MCV1 coverage exceeded 90% from 1990 to 2008 but dropped significantly to 42–79% between 2009 and 2016 (7), largely due to vaccine hesitancy stemming from reports of adverse events and vaccine supply shortages (11). This period of reduced vaccination coverage may have led to gaps in immunity among those who missed vaccinations during this time, particularly in younger cohorts. Although vaccination coverage rebounded to 88% by 2021 (7), the impact of prior disruptions in vaccination efforts may persist, contributing to the lower seroprevalence observed in Ukrainian immigrants.

Mongolia introduced MCV1 in 1973 with limited initial coverage (1–20%), and MCV2 in 1989 (7). Since the mid-1990s, MCV1 coverage has exceeded 95% (12). However, a large measles outbreak in 2015–2016 primarily affected young adults aged 15–24 years, revealing immunity gaps among those born before MCV2 introduction or missed during supplementary immunization activities (SIAs) (12). The low measles IgG seroprevalence observed in Mongolian immigrants in our study may reflect these historical immunity gaps.

In Cambodia, the measles monovalent vaccine was introduced into the routine immunization program in 1986. The first-dose coverage rate of measles-containing vaccine (MCV1) has shown an increasing trend since 2004, consistently achieving high coverage rates (above 90%) since 2009 and reaching 95% in 2015 (13). Since 2008, WHO and UNICEF Estimates of National immunization Coverage (WUENIC) have shown MCV1 coverage peaking at 92% in 2009 (7) but stabilizing at 83–87% in subsequent years, indicating consistently moderate vaccination rates over time. In our study, Cambodian immigrants showed a measles IgG positivity rate of 78.1%, which is consistent with Cambodia’s historical vaccination trends. Similarly, a previous study reported a measles IgG positivity rate of 79.2% among marriage migrants from Cambodia (4).

In this study, due to the limited reliability of vaccination histories collected through self-reporting, we could not directly analyze the relationship between vaccination status and antibody positivity. Previous studies have demonstrated that measles antibodies acquired through natural infection tend to persist for a longer duration compared to vaccine-induced immunity, which may wane over time, particularly in older age groups (14). As discussed earlier, the variability in measles IgG seropositivity observed among countries in this study can be attributed to multiple factors. In Ukraine, reduced vaccination coverage during certain periods likely contributed to immunity gaps. In Russia, increased vaccination coverage may have resulted in reduced natural immunity within the population, combined with waning immunity over time. Kazakhstan highlights potential discrepancies between reported vaccination rates and actual vaccination practices, as evidenced by gaps in documented vaccination histories observed during outbreaks. These factors collectively underscore the multifaceted nature of immunity dynamics and the importance of considering both natural infection and vaccination coverage when evaluating population immunity.

Notably, higher measles IgG positivity rates were observed among long-term residents, which included individuals on marriage visas, permanent residents, and naturalizations in present study. This finding is likely linked to the fact that 93 of the 98 long-term residents were women, a demographic more likely to have undergone rubella antibody testing as part of pre-pregnancy healthcare protocols. Women planning pregnancy are recommended to receive MMR vaccinations if they test negative for rubella antibodies (15), potentially explain the higher measles seropositivity in this group. This suggests that targeted testing and vaccination of immigrant women may contribute to higher immunity in females, although the difference was not statistically significant (*p* = 0.05).

This study has several limitations. First, the participants were primarily immigrants residing in Gwangju, South Korea, which may limit the generalizability of the findings to the broader immigrant population across the country. Additionally, the study included immigrants from a wide range of countries, and the sample size for each country was relatively small, which may reduce the statistical power when comparing country-specific antibody positivity rates.

Despite these limitations, the country-specific seroprevalence data presented in this study are valuable for estimating the potential risk of measles reintroduction in particular communities. These findings can inform public health strategies such as MMR vaccination recommendations and mandatory measles antibody testing for immigrants based on their country of origin. Targeted vaccination and screening programs for immigrants from countries with lower seroprevalence, such as Russia, Kazakhstan, and Ukraine, could play a crucial role in mitigating the risk of future outbreaks and safeguarding both immigrant and native populations in South Korea.

## Data Availability

The datasets presented in this study can be found in online repositories. The names of the repository/repositories and accession number(s) can be found at: https://doi.org/10.7910/DVN/632XJY.
