# Supplementary material for: Seroprevalence of measles antibody among immigrants in Gwangju, South Korea
Source: Front Public Health. 2024 Dec 19;12:1505489. doi: 10.3389/fpubh.2024.1505489 (PMC11694409; doi:10.3389/fpubh.2024.1505489)
Supplement: Supplementary file 2 [file Data_Sheet_2.PDF]

NO.			

신체계측	혈압	혈당	채혈	소변	설문	X-ray

광주광역시 거주 이주민 건강실태조사

안녕하십니까?

본 설문조사는 이주민의 건강실태와 의료이용 현황을 파악하여 앞으로 이주민의 건강권을 보장할 수 있는 정책 수립의 근거자료로 활용하고자 합니다.

응답하신 모든 내용은 통계법 제33조에 따라 연구 목적 이외에는 사용되지 않으며 비밀이 보장될 것을 약속드립니다.

모든 질문에 정확하고 솔직하게 응답 해주시기 바랍니다. 참여에 진심으로 감사드립니다.

2022년 9월

▶ 문의처 : 광주광역시 공공보건의료지원단 주임연구원 김진형 (070-4156-7446)

인적사항	이름		성별	남 / 여
	출생년도	년 월 일	최초 입국일자	년 월
	주소	구 동	연락처	
키/몸무게		cm/ kg	혈압(수축기/확장기)	/
공복시간		최근 식사시간 __시__분<br>_____시간째 공복	혈당	

1. 귀하의 국적은 어디입니까?

① 러시아 ② 우즈베키스탄 ③ 카자흐스탄
④ 우크라이나 ⑤ 투르크메니스탄 ⑥ 타지키스탄
⑦ 키르기스스탄 ⑧ 기타( )

2. 귀하는 현재 국민건강보험에 가입되어 있습니까?

① 건강보험 ② 의료급여
③ 입국 6개월 미만, 신청예정 ④ 보험료 미납으로 자격상실
⑤ 입국 6개월 경과, 신청안함

3. 귀하의 현재 체류자격은 무엇입니까?

① 재외동포 (F-4) ② 방문취업 (H-2) ③ 영주 (F-5)
④ 결혼이민 (F-6) ⑤ 방문동거 (F-1) ⑥ 거주 (F-2)
⑦ 비전문취업 (E-9) ⑧ 유학 (D-2) ⑨ 미등록
⑩ 기타

4. 귀하는 현재 어떤 업종에서 일을 하고 있습니까?

① 제조업 ② 건설업 ③ 농업·축산업
④ 어업·수산업 ⑤ 가사·간병 서비스업 ⑥ 음식·숙박 서비스업
⑦ 그 밖의 서비스업 ⑧ 학생 ⑨ 일을 하지 않고 있음
⑩ 기타 ( )

5. 지난 한 해 동안 귀하의 월 평균 소득은 얼마였습니까?

① 100만원 미만 ② 100만원 이상-150만원 미만 ③ 150만원 이상-250만원 미만
④ 250만원 이상-350만원 미만 ⑤ 350만원 이상 ⑥ 임금 또는 보수 없었음

6. 지금까지 살아오는 동안 피운 담배의 양은 총 얼마나 됩니까?

① 5갑(100개비) 미만
② 5갑(100개비) 이상
③ 피운 적 없다

7. 현재 담배를 피우고 계십니까?

① 매일 피운다 ② 가끔 피운다 ③ 금연했다 (현재 피우지 않음)

8. 평소에 본인의 건강은 어떻다고 생각합니까?

① 매우 좋음 ② 좋음 ③ 보통 ④ 나쁨 ⑤ 매우 나쁨

9. 병원에서 다음과 같은 질병을 진단받은 적이 있습니까? 진단받은 적이 있다면 진단받은 연도와 치료 여부를 체크해주세요.

질환	의사 진단 여부	진단연도	치료 여부
당뇨	①예 ②아니오	______년	①치료 중 ②치료중단 ③치료받은적 없음
고혈압	①예 ②아니오	______년	①치료 중 ②치료중단 ③치료받은적 없음
고지혈증	①예 ②아니오	______년	①치료 중 ②치료중단 ③치료받은적 없음
폐질환	①예 ②아니오	______년	①치료 중 ②치료중단 ③치료받은적 없음
신장질환	①예 ②아니오	______년	①치료 중 ②치료중단 ③치료받은적 없음
심장질환	①예 ②아니오	______년	①치료 중 ②치료중단 ③치료받은적 없음
뇌혈관질환	①예 ②아니오	______년	①치료 중 ②치료중단 ③치료받은적 없음
암질환	①예 ②아니오	______년	①치료 중 ②치료중단 ③치료받은적 없음
기타질환 (　　　　)	①예 ②아니오	______년	①치료 중 ②치료중단 ③치료받은적 없음

10. 병원에서 아래의 질환을 진단받은 적이 있다면 표시하여 주십시오.

질환	최근 진단된 연도	현재 치료 여부	치료완료의 경우
결핵	______년	①예 ②아니오 ③치료완료	치료기간:________개월
수두	______년	①예 ②아니오	
홍역	______년	①예 ②아니오	
B형간염	______년	①예 ②아니오	
C형간염	______년	①예 ②아니오 ③치료완료	치료기간:________개월
매독	______년	①예 ②아니오 ③치료완료	치료횟수: ________회
HIV 감염	______년	①예 ②아니오	

11. 아래 질환에 대한 예방접종을 받은 적이 있습니까?

질환	예방접종 여부	예방접종 횟수
결핵 예방접종(BCG)	①예 ②아니오 ③모름	
홍역(MMR 혹은 MR)	①예 ②아니오 ③모름	①1회 ②2회 ③모름
수두(varicella zoster)	①예 ②아니오 ③모름	①1회 ②2회 ③모름
B형 간염 예방접종	①예 ②아니오 ③모름	①1회 ②2회 ③3회 ④모름

12. 귀하는 한국에서 질병을 치료하기 위한 목적으로 수술을 받은 적이 있습니까?

① 있음 **(☞ 12-1 문항으로)**　② 받을 예정임 **(☞ 12-1 문항으로)**　③ 없음

12-1. 수술을 받은 적이 있거나 받을 예정인 경우 아래내용을 작성해주세요.

질병명	병원명	수술내용	치료비용(만원)

13. 귀하는 현재 가족(친척 포함)과 함께 살고 있습니까?

① 예 (동거가족 수______명)
② 가족 이외 동거인과 살고 있음 (동거인______명)
③ 아니오 (혼자 살고 있음)

14. 귀하는 같은 방을 사용중인 사람(가족 또는 친구)이 있습니까?

① 예 (______명)　② 아니오 (혼자 사용하고 있음)

15. 귀하는 지난 2년 이내에 국민건강보험공단에서 시행한 건강검진을 받은 적이 있습니까?

① 예　② 아니오

16. 귀하는 지난 2년 이내에 암검진을 받은 적이 있습니까?

(국가암검진, 국민건강보험공단 시행 암검진, 그 외 사비로 받은 검진 모두 포함)

① 예　② 아니오

17. 귀하는 지난 2년 간 아프거나 다쳤을 때 주로 어떻게 하셨습니까? (2개 선택)

① 개인병원에 갔다 ② 응급실이나 종합병원에 갔다
③ 보건소에 갔다 ④ 외국인 무료진료소에 갔다
⑤ 병/의원 처방없이 약국에 갔다 ⑥ 본국에서 가져온 약을 먹었다
⑦ 특별한 치료를 하지 않았다 ⑧ 민간요법으로 치료했다
⑨ 기타( )

18. 지난 2년간, 본인이 생각하기에 병의원(한방 포함) 치료 또는 검사를 받아 볼 필요가 있었으나 병의원에 가지 못한 적이 한 번이라도 있었습니까?
(단, 치과치료, 치과검사, 미용·성형 목적 진료 제외)

① 예, 가지 못한 적이 있다 (☞ **18-1 문항으로)**
② 아니요, 가지 못한 적이 없다
③ 병의원 치료 또는 검사가 필요한 적이 없었다

18-1. 지난 2년간, 필요한 병의원 치료나 검사를 받지 못하신 이유는 무엇입니까? 문항별 해당하는 경우 '예', 해당하지 않는 경우 '아니오'에 체크해주세요.

문항	예	아니오
1. 시간을 내기가 어려워서 (직장근무 때문에 병원갈 시간이 없다 등)	①	②
2. 비용이 부담 되어서 (의료비·교통비 부담, 일을 못해서 생기는 금전적 손실 부담 등)	①	②
3. 건강보험 미가입 또는 자격정지	①	②
4. 언어 소통이 되지 않아서	①	②
5. 교통편이 불편해서, 거리가 멀어서, 거동이 불편해서	①	②
6. 어느 병원 혹은 진료과에 가야 할지 몰라서	①	②
7. 기타 ( )		

19. 지난 2년간, 본인이 생각하기에 치과 치료 또는 검사를 받아 볼 필요가 있었으나 병의원에 가지 못한 적이 한 번이라도 있었습니까?
(단, 미백 치료 등 미용·성형 목적 진료 제외)

① 예, 가지 못한 적이 있다
② 아니요, 가지 못한 적이 없다
③ 병의원 치료 또는 검사가 필요한 적이 없었다

20. 본인의 건강관리를 위해 필요하다고 생각되는 것은 무엇입니까? (2개 선택)

① 건강보험 가입
② 의료비 지원
③ 무료진료 확대
④ 병원(약국) 방문 시 통역 지원
⑤ 한국 보건의료제도에 대한 정보 제공
⑥ 건강관리 상담 제공
⑦ 건강증진 활동 참여
⑧ 필수 예방접종 시행
⑨ 심리상담 제공
⑩ 기타( )

21. 평소 일상생활에서 스트레스를 어느 정도 느끼고 있으십니까?

① 대단히 많이 느낀다
② 많이 느끼는 편이다
③ 조금 느끼는 편이다
④ 거의 느끼지 않는다

22. 최근 1년 동안 2주 이상 연속으로 일상생활에 지장이 있을 정도로 슬프거나 절망감 등을 느낀 적이 있습니까?

① 있음 (☞ **22-1 문항으로)** ② 없음

22-1. 최근 1년 동안 정신건강 문제로 전문가와 상의하신 적이 있습니까?

① 있음 ② 없음
