# Supplementary material for: Seroprevalence of measles antibody among immigrants in Gwangju, South Korea
Source: Front Public Health. 2024 Dec 19;12:1505489. doi: 10.3389/fpubh.2024.1505489 (PMC11694409; doi:10.3389/fpubh.2024.1505489)
Supplement: Supplementary file 3 [file Data_Sheet_3.PDF]

NO.			

Physical	Blood pressure	Blood Sugar	Blood sample	Urine	Survey	X-ray	Translation Unavailable

Gwangju Metropolitan City Immigrant Health Status Survey

Hello,

This survey aims to understand the health status and medical usage of immigrants to develop policy foundations that can ensure the health rights of immigrants in the future.

All your responses will be kept confidential and will only be used for research purposes, as per Article 33 of the Statistics Law.

Please answer all questions accurately and sincerely.

Thank you for your participation.

2023. 10.

▶ Contact: Gwangju Metropolitan City Public Health and Medical Support Team Senior Researcher Kim Jeong-Yeon (070-4184-2194)

Personal Information	Name		Gender	M / F
	D.O.B		Date of first entry	
	Address		Contact	
Height/weight		cm/ kg	Blood Pressure (Systolic/Diastolic)	/
Fasting duration		Last meal __hour mins<br>____ hours fasting	Blood sugar	

1. What is your nationality?

① Vietnam ② China ③ Uzbekistan
④ Mongolia ⑤ Cambodia ⑥ Russia
⑦ Philippines ⑧ Nepal ⑨ Other ( )

2. Are you currently enrolled in National Health Insurance?

① Enrolled ② Not enrolled

3. What is your current residency status?

① Overseas Korean (F-4) ② Working Holiday (H-2) ③ Permanent Residency (F-5)
④ Marriage Immigration (F-6) ⑤ Tourist (F-1) ⑥ Residency (F-2)
⑦ Non-professional Employment (E-9) ⑧ Student Visa (D-2) ⑨ Unregistered
⑩ Other

4. What industry are you currently working in?

① Manufacturing ② Construction ③ Agriculture/Livestock
④ Fisheries/Marine ⑤ Housekeeping/Service ⑥ Food·Lodging
⑦ Other services ⑧ Student ⑨ Not working
⑩ Other ( )

5. What was your average monthly income over the past year?

① Less than 1 million won ② 1 million - less than 1.5 million won ③ 1.5 million - less than 2.5 million won
④ 2.5 million - less than 3.5 million won ⑤ 3.5 million won and above ⑥ No salary or wages

6. How many cigarettes have you smoked in your lifetime?

① Less than 5 packs (100 cigarettes)
② More than 5 packs (100 cigarettes)
③ Never smoked

7. Are you currently smoking?

① Smoke everyday ② Smoke occassionally ③ Quit smoking

8. How would you rate your overall health?

① Very Good ② Good ③ Average ④ Poor ⑤ Very poor

9. Have you ever been diagnosed with the following diseases by a doctor? If so, check the year of diagnosis and treatment status.

Disease	Diagnosis	Treatment
Diabetes	① Yes ② No	① Ongoing ② Stopped ③ Never treated
Hypertension	① Yes ② No	① Ongoing ② Stopped ③ Never treated
Hyperlipidemia	① Yes ② No	① Ongoing ② Stopped ③ Never treated
Other Diseases ( )	① Yes ② No	① Ongoing ② Stopped ③ Never treated

10. Have you ever been diagnosed with the following diseases by a hospital? If so, please mark accordingly.

Disease	Year of Diagnosis	Current Treatment Status	Completion Status
Tuberculosis		① Yes ② No ③ Completed	Duration of Treatment: ________months
Chickenpox		① Yes ② No	
Measles		① Yes ② No	
Hepatitis B		① Yes ② No	
Hepatitis C		① Yes ② No ③ Completed	Duration of Treatment: ________months
Syphilis		① Yes ② No ③ Completed	Duration of Treatment: ________months
HIV Infection		① Yes ② No	

11. Have you ever received vaccinations for the following diseases?

Disease	Vaccination Status	Number of Doses
BCG (Tuberculosis)	① Yes ② No ③Don't Know	
Measles (MMR or MR)	① Yes ② No ③Don't Know	① 1 ② 2 ③ Don't Know
Chickenpox (varicella zoster)	① Yes ② No ③Don't Know	① 1 ② 2 ③ Don't know
Hepatitis B vaccination	① Yes ② No ③Don't Know	① 1 ② 2 ③ 3 ④ Don't know

12. Have you received a health check-up conducted by the National Health Insurance Service in the last 2 years?

① Yes ② No

13. Have you received a cancer screening in the last 2 years?

(This includes national cancer screenings, screenings conducted by the National Health Insurance Service, and any screenings received privately)

① Yes ② No

14. What did you primarily do when you were sick or injured in the past 2 years? (Select 2)

① Went to a private hospital
② Went to the ER or a general hospital
③ Went to a public health center
④ Went to a free clinic for foreigners
⑤ Went to a pharmacy without a doctor's prescription
⑥ Took medicine brought from my home country
⑦ Did not receive any special treatment
⑧ Treated with traditional remedies
⑨ Others ( )

15. In the past year, have you ever felt the need to visit a doctor (including traditional Korean medicine) for treatment or examination, but could not? (Excluding dental care, cosmetic surgery)

① Yes, I couldn't go (Proceed to question 15−1)
② No, I could go
③ There was no need for treatment or examination

15-1. What is the reason you could not receive the necessary medical treatment or

examination in the past year? Please check 'Yes' for applicable reasons and 'No' for non-applicable ones.

Case	Yes	No
1. Difficulty in sparing time (e.g., no time due to work)	①	②
2. Financial burden (e.g., medical fees, transportation costs)	①	②
3. Not covered by health insurance or eligibility suspended	①	②
4. Language barriers	①	②
5. Transportation inconvenience, long distance, or physical difficulty	①	②
6. Uncertainty about which hospital or department to visit	①	②
7. Others ( )		

16. In the past year, have you ever felt the need for dental care or examination, but could not go to a clinic? (Excluding cosmetic treatments)

① Yes, I couldn't go
② No, I could go
③ There was no need for treatment or examination

17. What do you think is necessary for your health management? (Select 2)

① Joining health insurance
② Medical cost support
③ Expansion of free medical services
④ Interpretation support during hospital/clinic visits
⑤ Providing information about the Korean healthcare system
⑥ Providing health management consultations
⑦ Participation in health promotion activities
⑧ Implementing essential vaccinations
⑨ Providing psychological counseling
⑩ Others ( )

18. How much stress do you generally feel in your daily life?

① Extremely high
② Quite high
③ Somewhat
④ Almost none

19. In the past year, have you felt sadness or despair that interfered with your daily life for more than two weeks consecutively?

① Yes (Proceed to question 19-1) ② No

19-1. Have you consulted with a professional about mental health issues in the past year?

① Yes ② No
