# Supplementary figures and images for: Seroprevalence of measles antibody among immigrants in Gwangju, South Korea
Source: Front Public Health. 2024 Dec 19;12:1505489. doi: 10.3389/fpubh.2024.1505489 (PMC11694409; doi:10.3389/fpubh.2024.1505489)

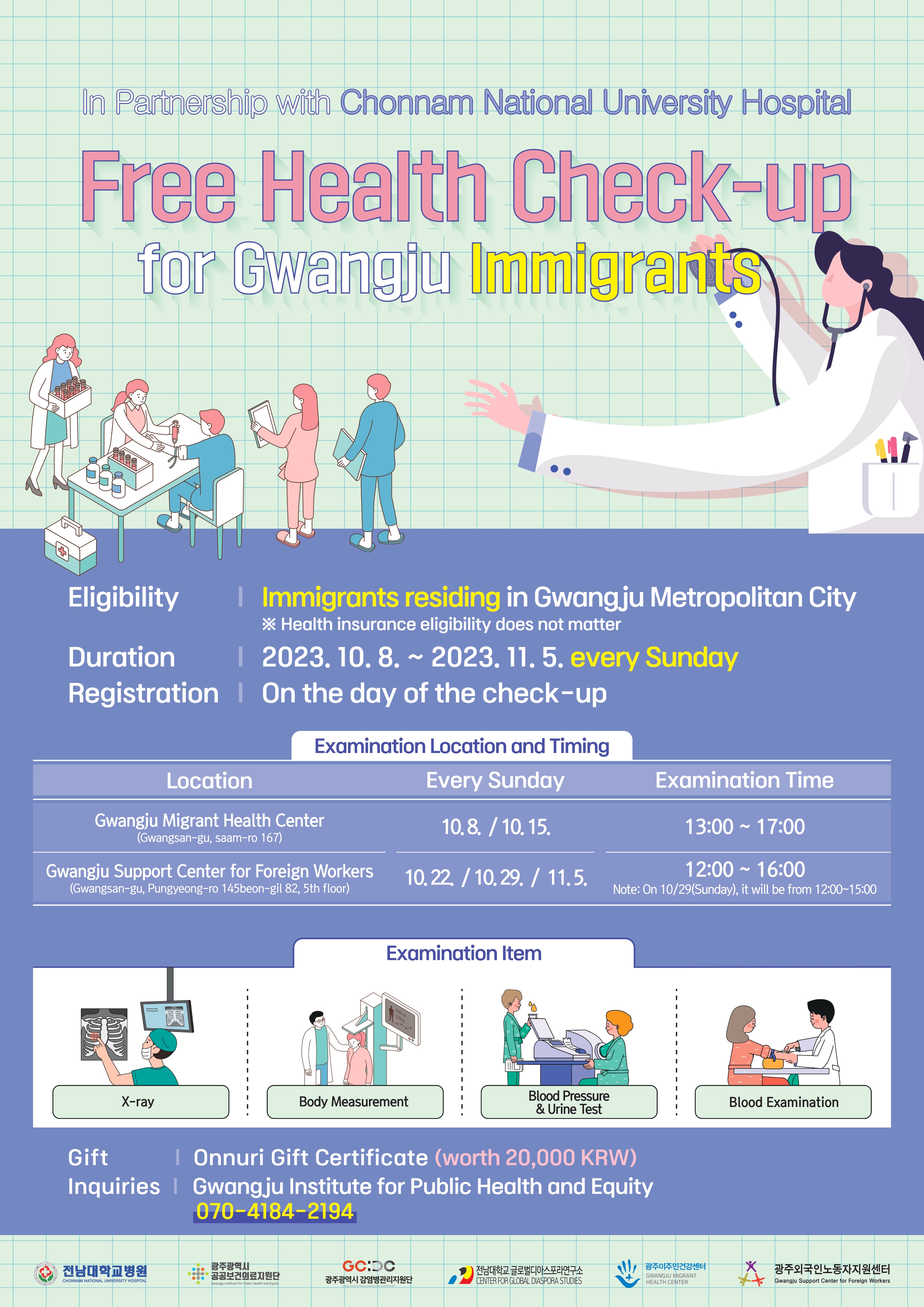

Supplement: Supplementary file 4 [file Image_1.JPEG]

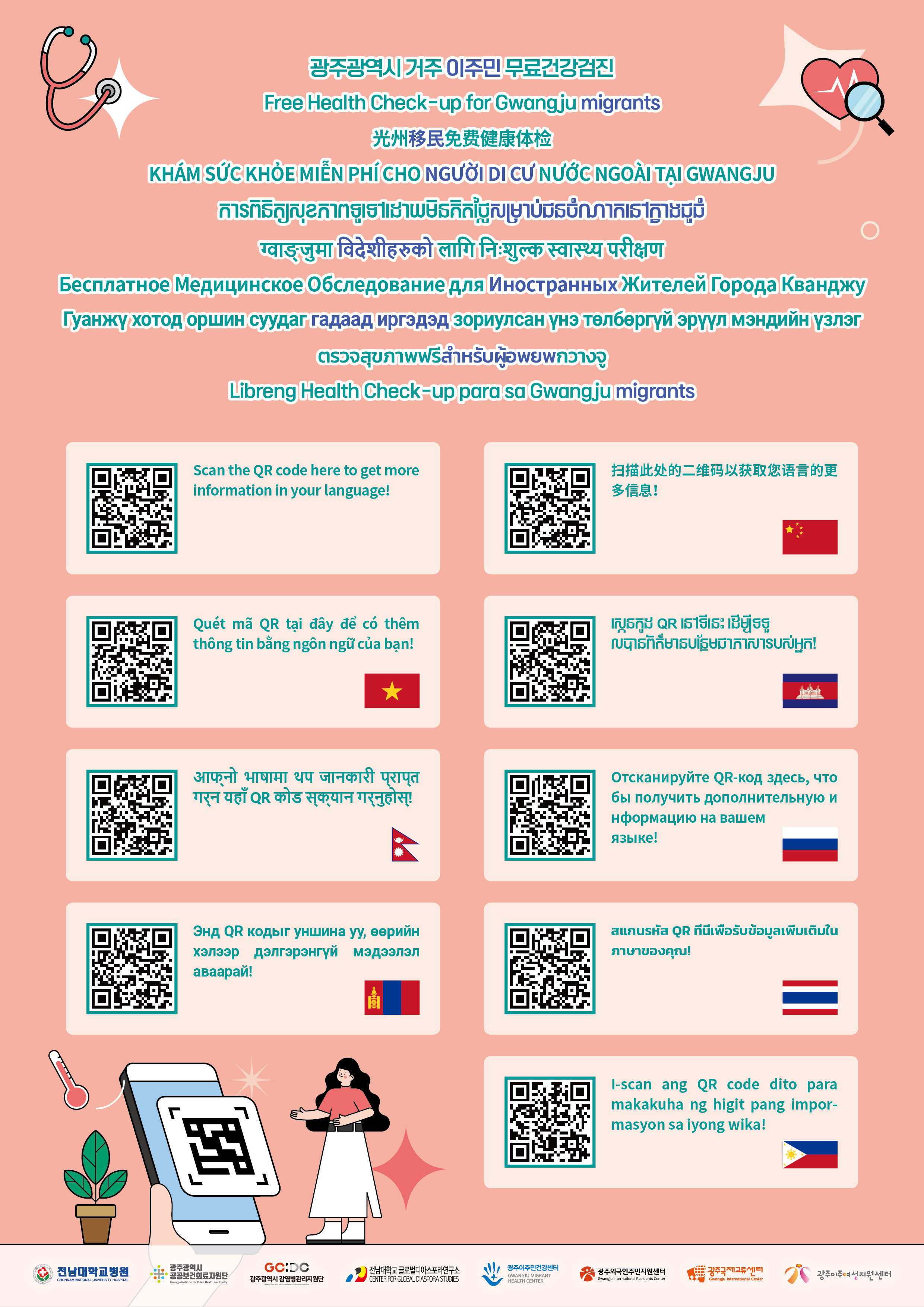

Supplement: Supplementary file 5 [file Image_2.JPEG]
